# Associations between serum amyloid A, interleukin‐6, and COVID‐19: A cross‐sectional study

**DOI:** 10.1002/jcla.23527

**Published:** 2020-08-28

**Authors:** Qian Liu, Yaping Dai, Meimei Feng, Xu Wang, Wei Liang, Fumeng Yang

**Affiliations:** ^1^ Department of Laboratory Medicine The Second People's Hospital of Lianyungang Lianyungang China; ^2^ Department of Laboratory Medicine Wuxi Fifth People's Hospital Wuxi China; ^3^ Department of General Pediatrics Zaozhuang Maternal and Child Health Hospital Zaozhuang China

**Keywords:** coronavirus disease, interleukin‐6, serum amyloid A, severe acute respiratory syndrome coronavirus 2

## Abstract

**Background:**

Serum amyloid A (SAA), interleukin‐6 (IL‐6) and neutrophil‐to‐lymphocyte ratio (NLR) play critical roles in inflammation and are used in clinical laboratories as indicators of inflammation‐related diseases. We aimed to provide potential laboratory basis for auxiliary distinguishing coronavirus disease (COVID‐19) by monitoring above indicators.

**Methods:**

A total of 84 patients with confirmed COVID‐19 were enrolled in the study. Baseline characteristics and laboratory results were collected and analyzed. Receiver operating characteristic (ROC) curve analysis was used to combined detection of SAA and IL‐6 in patients with COVID‐19, and independent risk factors for severity of COVID‐19 were assessed by using binary logistic regression.

**Results:**

The main clinical symptoms of patients with COVID‐19 were fever (98.8%), fatigue (61.9%), and dry cough (58.3%). SAA, IL‐6, and NLR were significantly higher in patients with COVID‐19 (all *P* < .001), and compared with nonsevere patients, three indicators of severe patients were significantly elevated. Besides, combined detection of SAA and IL‐6 better separates healthy people from patients with COVID‐19 than detection of SAA or IL‐6 alone. In addition, elevated SAA, IL‐6, and NLR can be used as independent variables for predicting the severity of patients with COVID‐19.

**Conclusion:**

Serum amyloid A and IL‐6 could be used as addition parameters to helping the distinguish of patients with COVID‐19 from healthy people, and can provide potential basis for separating patients with nonsevere and severe clinical signs.

## Graphical abstract

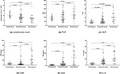

**Abbreviations**
ARDS: acute respiratory distress syndrome
COVID‐19: coronavirus disease
CRP: C‐reactive protein
IQR: interquartile range
MERS: Middle East respiratory syndrome
NLR: neutrophil‐to‐lymphocyte ratio
OR: odds ratio
PLR: platelet‐to‐lymphocyte ratio
ROC: receiver operating characteristic
SAA: serum amyloid A; IL‐6: interleukin‐6
SARS: severe acute respiratory syndrome
SARS‐CoV‐2: severe acute respiratory syndrome coronavirus 2
WHO: World Health Organization

## 1 INTRODUCTION

Coronaviruses mainly cause respiratory infections and some strains, such as severe acute respiratory syndrome (SARS) and Middle East respiratory syndrome (MERS), are associated with high infectivity and mortality, and thus are harmful to public health.^1^, ^2^ In December 2019, pneumonia in people infected with severe acute respiratory syndrome coronavirus 2 (SARS‐CoV‐2) appeared in Wuhan, Hubei Province. The infectious disease has since spread to other parts of the country and many overseas countries.^3^, ^4^, ^5^ According to the official website of the Chinese Center for Disease Control and Prevention (http://2019ncov.chinacdc.cn/nCoV/), as of March 11, 2020, at 10:00, China had cumulatively diagnosed approximately 80 955 patients with coronavirus disease (COVID‐19), and other countries have accumulated approximately 36,900 cases. Basic, clinical, and epidemiological research on COVID‐19 continues to be reported.^6^, ^7^, ^8^, ^9^

Existing reports show that approximately 84% of patients were categorized as nonsevere cases based on clinical symptoms, such as fever, cough, myalgia, or fatigue.^10^, ^11^, ^12^ The rest of others were categorized as severe or critical cases which were mainly accompanied by acute respiratory distress syndrome (ARDS) or acute respiratory failure, and the median time from onset of symptoms to ARDS was about 9 days.^10^, ^11^, ^12^ Besides, different types of patients often needed different plans of care, such as isolation for mild patients and intensive care units (ICU) for severe cases. Therefore, it is important to identify the risk factors of COVID‐19 as early as possible so as to take appropriate interventions. Previous studies indicated that serum amyloid A (SAA) and interleukin‐6 (IL‐6) play important roles in viral diseases and were widely used in clinical laboratories as indicators of inflammation.^13^, ^14^, ^15^, ^16^, ^17^ So far, the cytokine profile including IL‐2, IL‐6, IL‐7, granulocyte colony‐stimulating factor, interferon‐γ, inducible protein 10, monocyte chemoattractant protein 1, macrophage inflammatory protein 1‐α, and tumor necrosis factor‐α has been considered to be associated with the severity of COVID‐19.^18^ In view of the above considerations, we aimed to provide potential laboratory basis for auxiliary distinguishing coronavirus disease (COVID‐19) by detecting inflammation‐related markers of SAA and IL‐6.

## 2 METHODS

### 2.1 Patients and inclusion criteria

A total of 84 patients confirmed COVID‐19 admitted to the Second People's Hospital of Lianyungan and Wuxi Fifth People's Hospital from January 23, 2020, to February 29, 2020, were enrolled. The diagnosis of COVID‐19 was based on WHO interim guidance^19^ and confirmed by laboratory test for SARS‐CoV‐2 from the respiratory specimens show positive result by the real‐time reverse transcription–polymerase chain reaction (RT‐PCR) assay. But patients with acute trauma, autoimmune diseases, except respiratory tract infectious diseases, and hematological diseases having undergone radiotherapy and chemotherapy were excluded.

According to the novel coronavirus pneumonia diagnosis and treatment plan (trial version 7),^20^ the clinical classification of COVID‐19 was as follows: (a) mild type: mild clinical symptoms, no typical changes in lungs on CT imaging; (b) regular type: fever, respiratory tract symptoms and CT imaging showing typical changes in lungs, such as multiple small patch shadows, interstitial changes, multiple ground glass shadows, infiltration shadows, and pulmonary consolidation; (c) severe type: respiratory distress (respiratory rate ≥30 breaths/min), mean oxygen saturation ≤93% in resting state or arterial blood oxygen partial pressure/oxygen concentration ≤ 300 mm Hg (1 mm Hg = 0.133 kPa); (d) critical type: respiratory failure requiring mechanical ventilation, shock, or ICU admission for combined organ failure. On the basis of the above classification, we divided all patients into two groups: (1) nonsevere, including mild and regular types, and (2) severe, including severe and critical types. Meanwhile, 30 healthy subjects (15 males and 15 females) were selected as a control group, and above subjects were confirmed with the negative results of SARS‐CoV‐2 by using the method of real‐time RT‐PCR. This study was approved by the Ethics Committee of the Second People's Hospital of Lianyungan, and all participants provided signed informed consent.

### 2.2 Instruments and reagents

The white blood cell count, neutrophil count, lymphocyte count, red blood cell count, platelet count, and hemoglobin and C‐reactive protein (CRP) levels were determined with a BC‐5390 automatic hematology analyzer and associated reagents (Mindray Co., Ltd.). SAA was determined with commercially available assay kits (lot: 20190815, Zhuoyun Biotechnology Co., Ltd.) and an automatic biochemical analyzer (Beckman Co., Ltd.) by using the method of latex immunoturbidimetry. IL‐6 was measured with a commercially available assay kit (lot: 921324, Beckman Co., Ltd.) and an Access 2 automatic immune analysis system (Beckman Co., Ltd.) by using the method of enzyme‐linked immunochemiluminescence. All equipment was maintained and calibrated according to the requirements. Internal quality control and external quality assessment were performed.

### 2.3 Specimen collection

From all subjects, 2 mL of EDTA K_2_ anticoagulated venous blood was collected on admission for detection of the white blood cell count, neutrophil count, lymphocyte count, red blood cell count, platelet count, and hemoglobin and CRP levels. Meanwhile, 5 mL of venous blood (with a separation tube) was collected for the detection of SAA and IL‐6. The latter specimens were centrifuged at 1200 × *g* for 10 minutes, and the detection of all analytes was completed within 4 hours.

### 2.4 Statistical analyses

Data were analyzed in IBM SPSS Statistics 21.0, and data are expressed as median with interquartile range (IQR). The Mann‐Whitney test was used to compare two independent groups. The Kruskal‐Wallis test was used to compare multiple groups, and Dunnett's test was used for pairwise comparisons. Categorical variables were analyzed with chi‐square test. The combined detection of laboratory indicators was analyzed with the receiver operating characteristic (ROC) curve, and risk factors were evaluated with binary logistic regression. A *P* value <.05 was considered statistically significant.

## 3 RESULTS

### 3.1 Baseline characteristics of patients with COVID‐19

The study included 84 hospitalized patients with COVID‐19:59 were placed in the nonsevere group (mild and regular type), and 25 were placed in the severe group (severe and critical type) on admission. There was no statistical difference in the sex ratio, age, body mass index (BMI), and smoking status between the nonsevere group and severe group (*P* > .05). The median blood pressure (systolic pressure and diastolic pressure) in the two groups was significantly different (*P* < .05). Both systolic and diastolic blood pressure were significantly higher in the severe group than in the nonsevere group. Among the 84 patients, 83 (98.8%) had fever, 52 (61.9%) had fatigue, 49 (58.3%) had dry cough, 31 (36.9%) had myalgia, 26 (31.0%) had headache, 16 (19.0%) had expectoration, 14 (16.7%) had pharyngalgia, 10 (11.9%) had diarrhea, and eight (9.5%) had nausea or vomiting; however, there was no statistical difference between the nonsevere group and severe group (*P* > .05). Of the 84 patients with COVID‐19, 33 (39.3%) had anorexia, and 23 (27.4%) had dyspnea; there was a statistical difference between the two groups (*P* < .05). Among the 84 patients, five (6.0%) had diabetes, two (2.4%) had cardiovascular disease, four (4.8%) had cerebrovascular disease, three (3.6%) hadchronic kidney disease, five (6.0%) had chronic obstructive pulmonary disease, and one (1.2%) patient had cancer; however, there was no statistical difference between the nonsevere group and severe group (*P* > .05) (Table 1).

**Table 1:** Baseline characteristics of patients with COVID‐19 [median (IQR)]

Characteristic	All patients n = 84	Nonsevere group n = 59	Severe group n = 25	*P* value
Sex (male/female)	45/39	31/28	14/11	.8147
Age, median (IQR), y	51 (37‐59)	49 (33‐57)	52 (45‐67)	.0716
BMI, median (IQR), kg/m^2^	23.6 (22.3‐24.6)	23.4 (22.1‐24.3)	24.3 (22.9‐25.0)	.2213
Smoking status (yes/no)	7/77	4/55	3/22	.4197
Systolic pressure, median (IQR), mm Hg	129 (123‐138)	126 (122‐136)	136 (128‐141)	.0018
Diastolic pressure, median (IQR), mm Hg	80 (76‐83)	78 (74‐80)	84 (80‐89)	<.0001
Clinical symptoms				
Fever, no. (%)	83 (98.8)	58 (98.3)	25 (100)	1.0000
Fatigue, no. (%)	52 (61.9)	34 (57.6)	18 (72.0)	.3257
Dry cough, no. (%)	49 (58.3)	35 (59.3)	14 (56.0)	.8123
Myalgia, no. (%)	31 (36.9)	21 (35.6)	10 (40.0)	.8058
Anorexia, no. (%)	33 (39.3)	17 (28.8)	16 (64.0)	.0035
Headache, no. (%)	26 (31.0)	17 (28.8)	9(36.0)	.6076
Dyspnea, No. (%)	23 (27.4)	8 (13.6)	15 (60.8)	<.0001
Expectoration, no. (%)	16 (19.0)	11 (18.6)	5 (20.0)	1.0000
Pharyngalgia, no. (%)	14 (16.7)	6 (10.2)	8 (32.0)	.2901
Diarrhea, no. (%)	10 (11.9)	6 (10.2)	4 (16.0)	.4749
Nausea or vomiting, no. (%)	8 (9.5)	5 (8.5)	3 (12.0)	.6899
Coexisting disorder				
Diabetes, no. (%)	5 (6.0)	2 (3.4)	3 (12.0)	.1534
Cardiovascular disease, no. (%)	2 (2.4)	0 (0)	2 (8.0)	.0861
Cerebrovascular disease, no. (%)	4 (4.8)	1 (1.7)	3 (12.0)	.0769
Chronic kidney disease, No. (%)	3 (3.6)	2 (3.4)	1 (4.0)	1.0000
Chronic obstructive pulmonary disease, no. (%)	5 (6.0)	2 (3.4)	3 (12.0)	.1534
Cancer, no. (%)	1 (1.2)	0 (0)	1 (4.0)	.2976

Abbreviations: BMI, body mass index; IQR, interquartile range.

### 3.2 Laboratory indicators of patients with COVID‐19

**FIGURE 1:**
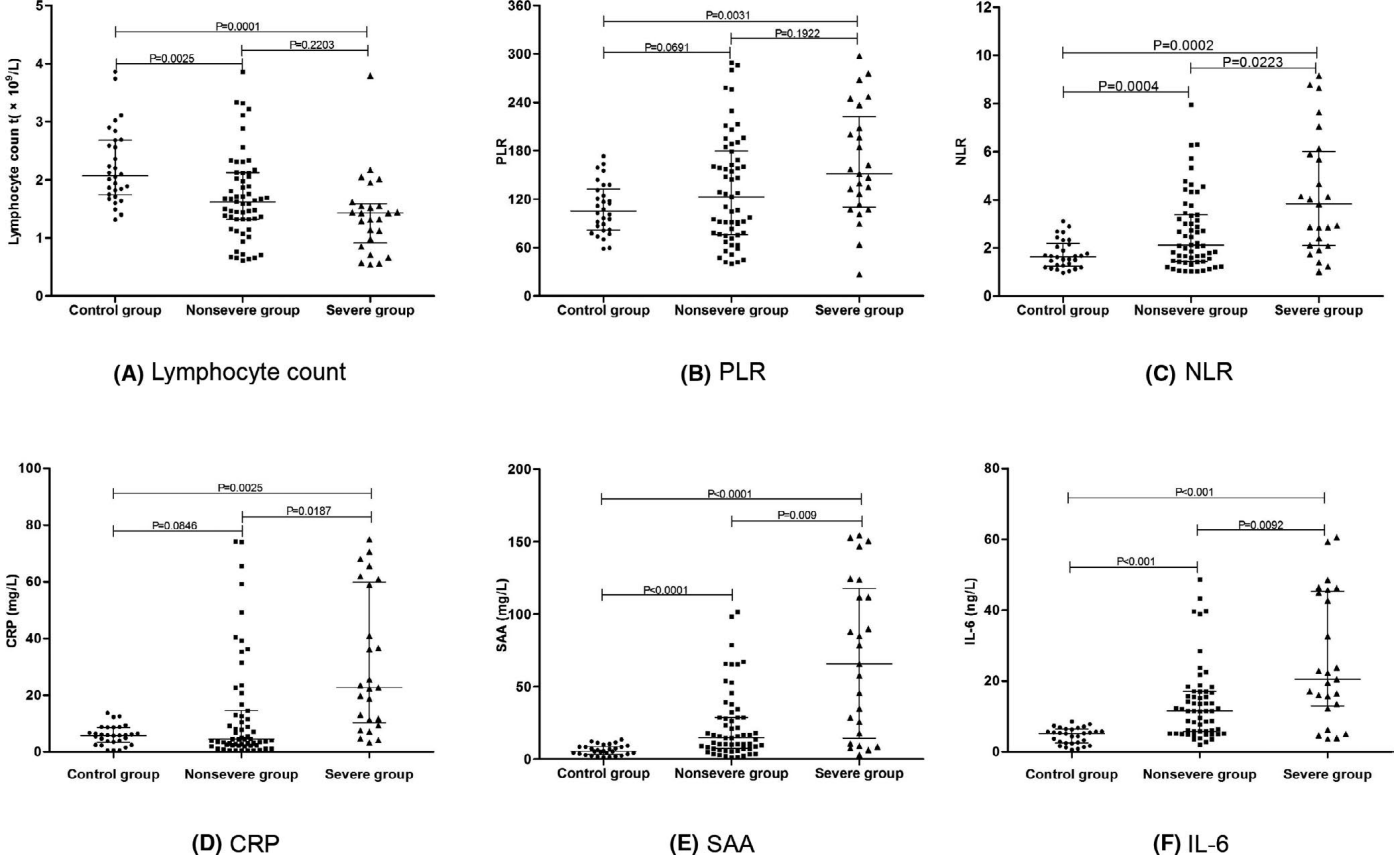
Comparison of laboratory indicators among patients with COVID‐19. Scatter‐dot plots showing the levels of Lymphocyte count (A), PLR (B), NLR (C), CRP (D), SAA (E), and IL‐6 (F) among control group, nonsevere group, and severe group. Error bars in the scatter‐dot plots indicate the median and interquartile ranges.

A significant difference was observed in lymphocyte count between patients with COVID‐19 and healthy individuals. Lymphocyte count was significantly lower in patients with COVID‐19, and it also showed a significant decrease in mild type patients (included in nonsevere group), but there was no statistical difference between the nonsevere group and the severe group. The levels of CRP were significantly higher in patients with severe disease than healthy controls and patients with nonsevere disease (*P* < .05). Moreover, the neutrophil‐to‐lymphocyte ratio (NLR), SAA, and IL‐6 were significantly higher in patients with COVID‐19 than in the healthy control group. In addition, the NLR, SAA, and IL‐6 in patients were significantly higher in the severe group than in the nonsevere group (*P* < .05). Compared with healthy controls and nonsevere group, platelet‐to lymphocyte‐ratio (PLR) was significantly increased in severe group (*P* < .05). However, the white blood cell count, neutrophil count, red blood cell count, platelet count, and hemoglobin were not statistically different between healthy controls and patients with COVID‐19 (Table 2, Figure 1).

**Table 2:** Laboratory indicators of patients with COVID‐19 [median (IQR)]

Indicators	Control group n = 30	Nonsevere group n = 59	Severe group n = 25	*P* value
White blood cell count, ×10^9^/L	6.35 (5.20‐7.70)	5.59 (4.66‐7.50)	6.44 (5.30‐8.24)	.2413
Neutrophil count, ×10^9^/L	3.63 (2.87‐4.42)	3.56 (3.06‐4.34)	4.27 (3.00‐6.31)	.1078
Lymphocyte count, ×10^9^/L	2.07 (1.74‐2.68)	1.62 (1.32‐2.12)	1.43 (0.92‐1.59)	<.0001
Red blood cell count, ×10^12^/L	4.83 (4.43‐5.13)	4.97 (4.42‐5.20)	4.55 (4.19‐4.92)	.0634
Hemoglobin, g/L	149 (136‐161)	147 (134‐154)	138 (126‐147)	.0642
Platelet count, ×10^9^/L	231 (190‐275)	200 (161‐243)	194 (141‐253)	.0734
PLR	105.20 (81.66‐132.70)	122.70 (76.42‐179.50)	151.30 (110.30‐22.60)	.0091
NLR	1.64 (1.25‐2.20)	2.11 (1.45‐3.38)	3.83 (2.10‐6.02)	<.0001
CRP, mg/L	5.80 (3.23‐8.60)	4.52 (2.30‐14.56)	22.74 (10.32‐63.24)	<.0001
SAA, mg/L	5.50 (3.33‐8.78)	14.70 (7.43‐28.69)	65.75 (14.30‐117.80)	<.0001
IL‐6, ng/L	5.17 (2.42‐6.37)	11.56 (5.77‐17.02)	20.50 (12.86‐45.30)	<.0001

Abbreviations: CRP, C‐reactive protein; IL‐6, interleukin‐6; IQR, interquartile range; NLR, neutrophil‐to‐lymphocyte ratio; PLR, platelet‐to‐lymphocyte ratio; SAA, amyloid A.

### 3.3 Combined detection of SAA and IL‐6 in patients with COVID‐19

**FIGURE 2:**
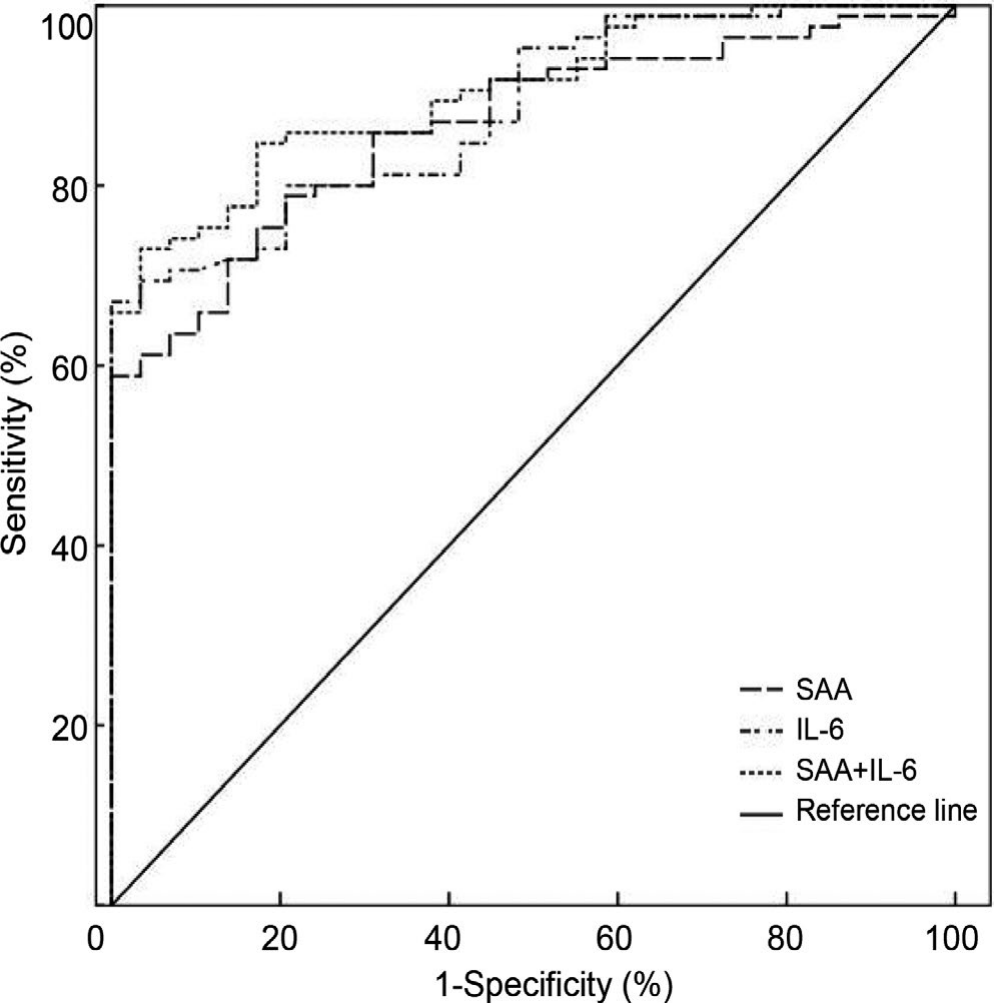
ROC curve of serum SAA and IL‐6 for patients with COVID‐19. When the detection of SAA alone, the area under the ROC curve was 0.865 (95% CI: 0.799‐0.931; *P* < .05). When the detection of IL‐6, the area under alone, the ROC curve was 0.882 (95% CI: 0.821‐0.943; *P* < .05). When the combined detection of them, the area under the ROC curve was 0.904 (95% CI: 0.851‐0.958; *P* < .05).

Based on the detection of nucleic acid as the gold standard for diagnosing COVID‐19, and compared with SAA and IL‐6, the ROC curve analysis showed that combined detection of SAA and IL‐6 better separates healthy people from patients with COVID‐19 than detection of SAA or IL‐6 alone (Figure 2).

### 3.4 Independent risk factors for severity of COVID‐19

All patients with COVID‐19 (nonsevere and severe group) were considered as dependent variables, and age, body mass index (BMI), smoking status, coexisting disorder, white blood cell count, neutrophil count, lymphocyte count, NLR, PLR, CRP, SAA, and IL‐6 were considered the independent variables. Binary logistic regression analysis showed that one unit increase in SAA, IL‐6, and NLR, the risk of nonsevere patients with COVID‐19 becoming severe patients increased significantly (SAA: OR = 4.212, *P* = .039; IL‐6: OR = 1.281, *P* = .029; NLR: OR = 5.180, *P* = .033). Therefore, our study indicated that SAA, IL‐6, and NLR can be used as independent variables for predicting the severity of patients with COVID‐19 (Table 3).

**Table 3:** Binary regression Analysis of risk factors for severity of COVID‐19

Independent variable	B	SE	Wals *χ* ^2^	*P* value	OR (95% CI)
Age	−0.284	0.452	0.395	.530	0.753 (0.310‐1.826)
BMI	−0.044	0.606	0.005	.942	0.957 (0.292‐3.138)
Blood pressure	−1.241	1.302	0.909	.341	0.289 (0.022‐3.711)
Smoking history	−0.429	1.409	0.093	.761	0.651 (0.041‐10.311)
Coexisting disorder	1.766	1.237	2.040	.153	5.850 (0.518‐66.053)
White blood cell count	−0.517	0.425	1.482	.223	0.596 (0.259‐1.371)
Neutrophil count	0.034	0.340	0.010	.920	1.035 (0.532‐2.014)
Lymphocyte count	1.366	1.009	1.834	.176	3.919 (0.543‐28.295)
NLR	1.645	0.772	4.537	.033	5.180 (1.140‐23.533)
PLR	−0.004	0.011	0.137	.711	0.996 (0.974‐1.018)
CRP	0.072	0.070	1.047	.306	1.074 (0.937‐1.232)
SAA	1.438	0.695	4.278	.039	4.212 (1.078‐16.453)
IL‐6	0.247	0.113	4.761	.029	1.281 (1.026‐1.600)
Constant	−6.443	4.450	2.096	.148	0.002

Abbreviations: B, regression coefficients; BMI, body mass index; CI, confidence interval; CRP, C‐reactive protein; IL‐6, interleukin‐6; NLR, neutrophil‐to‐lymphocyte ratio; OR, odds ratio; PLR, platelet‐to‐lymphocyte ratio; SAA, amyloid A; SE, standard error.

## 4 DISCUSSION

SARS‐CoV‐2 was discovered because of viral pneumonia cases in Wuhan in December 2019 and was named by the WHO on January 12, 2020. It is the same genus as SARS‐CoV and MERS‐CoV,^21^ and is extremely contagious, such that all people are susceptible. It is a cluster infectious disease and an outbreak occurred in the hospital. In severe cases, it can cause acute respiratory distress syndrome, multiple organ dysfunction, and even death.^22^, ^23^ In this study, we analyzed the clinical characteristics of 84 patients with COVID‐19. Most patients had respiratory symptoms such as fever and dry cough, as well as non‐respiratory symptoms such as fatigue, myalgia, anorexia, and headache. A retrospective analysis of the clinical characteristics of 138 patients with COVID‐19 in the Wuhan area by Wang et al^24^ has shown that fever, fatigue, cough, anorexia, myalgia, and dyspnea are the main clinical manifestations of patients with COVID‐19. Li et al^25^ have conducted a meta‐analysis of the clinical characteristics of patients with COVID‐19 and reported that the main clinical symptoms of patients with COVID‐19 are fever (88.5%), cough (68.6%), myalgia or fatigue (35.8%), expectoration (28.2%), and dyspnea (21.9%). The above research findings are essentially consistent with those from this study. Therefore, clinicians must place importance on the clinical symptoms of patients with suspected SARS‐CoV‐2 infection and improve their ability to distinguish SARS‐CoV‐2 infection from other conditions. Especially when the clinical manifestations are non‐respiratory symptoms, combined laboratory indicators and imaging findings should be used to diagnose patients as soon as possible, to enable rapid intervention and prevent the transmission of SARS‐CoV‐2.

According to the novel coronavirus pneumonia diagnosis and treatment plan (trial version 7),^20^ we divided 84 patients into a nonsevere group and a severe group. Retrospective analysis indicated that the blood pressure of patients with new severe disease was higher than that of patients with nonsevere disease, and there was a statistically significant difference between the two groups. The incidence of anorexia and dyspnea in patients with severe disease was significantly higher than that in those with nonsevere disease, and there was a statistically significant difference between the two groups. In addition, some patients still had some underlying diseases, such as diabetes, cardiovascular disease, cerebrovascular disease, chronic kidney disease, chronic obstructive pulmonary disease, or tumors, but there was no statistical difference in incidence between the two groups. A multicenter survey conducted by Guan et al^11^ has shown that 23.7% of patients with COVID‐19 have comorbidities, but the incidence of coexisting diseases was not statistically different between nonsevere and severe cases. Liu et al^26^ have conducted a survey of 137 patients with COVID‐19 and found that 19.7% of patients had coexisting diseases such as diabetes, cardiovascular disease, or hypertension, but the study did not compare the incidence of coexisting diseases between a nonsevere group and severe group. Although the above findings are consistent with those of this study, because the proportion of patients with COVID‐19 with comorbidities is small, the current data cannot confirm whether there is a link between the severity of COVID‐19 and comorbidities.

We analyzed the results of laboratory indicators and found that the white blood cell count and neutrophil count of the patients with COVID‐19 did not significantly differ, but the lymphocyte count was significantly lower than that in the healthy group. Moreover, the NLR of patients with COVID‐19 was significantly higher than that of the healthy group, and it was higher in patients with severe disease. A study conducted by Liu et al^27^ has shown that NLR is closely associated with the occurrence and progress of COVID‐19, and it can be used as an important indicator of the risk stratification of COVID‐19, thus supporting clinical adoption of appropriate treatment plans. Meanwhile, our study also indicated that PLR was significantly higher in severe patients than that of healthy group and nonsevere patients. One research conducted by Qu et al^28^ has demonstrated that the PLR of patients means the degree of cytokine storm, which might be associated with the prognosis in patient with COVID‐19. We also analyzed the changes in inflammation indicators such as CRP, SAA, and IL‐6 in patients with COVID‐19. We found that the levels of CRP were significantly higher in severe cases than in nonsevere cases, but the CRP levels were not statistically different between patients with nonsevere disease and healthy people. In addition, the serum SAA and IL‐6 levels of patients with COVID‐19 were significantly higher than those of healthy people, and both of them were higher with severe disease. A study conducted by Zhang et al^29^ have shown that SAA levels were increased in all patients with COVID‐19, with statistically significant differences between those with severe and mild cases. Besides, another research conducted by Zhang et al^30^ also demonstrated that elevatory range of SAA were associated with the degree of COVID‐19 severity, and SAA changes were greater than CRP, lymphocyte count, and neutrophil count. The above results were similar to our study and confirmed that the increased level of SAA was related to the severity of COVID‐19.

Through the combined detection of SAA and IL‐6 in patients, we also found that combined detection of the two can significantly improve the distinguishing of patients with COVID‐19 from healthy people. In addition, we further analyzed the independent variables for predicting the severity of COVID‐19. And we found that patients with higher levels of SAA, IL‐6, and NLR have greater risk to develop severe disease. Chen et al^31^ have conducted a study on serum cytokines in 29 patients with COVID‐19 and have found significantly elevated levels of serum IL‐6 in patients with COVID‐19, a result closely associated with the severity of the disease. One retrospective, multicenter research conducted by Ruan et al^32^ showed that non‐survivors had significantly higher levels of IL‐6 compared with survivors with COVID‐19, which suggested that mortality might be due to virally driven hyperinflammation. Evenmore, a multicenter, randomized controlled trial of tocilizumab (IL‐6 receptor blockade, licensed for cytokine release syndrome), has been approved in patients with COVID‐19 and elevated IL‐6 in China (ChiCTR2000029765).^33^ Similar to the above study, our research shows that IL‐6 and inflammation indicators are significantly elevated in patients with COVID‐19 and reflect the severity of the disease. Therefore, our study suggests that the expression levels of SAA and IL‐6 in patients with COVID‐19 should be detected as soon as possible, to provide an important laboratory basis for evaluating the severity of the disease.

There are some limitations to this study. Firstly, because only 84 patients with COVID‐19 were included, our conclusions must be further verified in more participants. Secondly, as patients come from different hospitals or regions, we have some difficulties in obtaining patient's data, which leads to incomplete acquire to some data, such as average time between infection and occurence of symptoms, the time from admission to the hospital and developing severe type, and part of laboratory indicators. Thirdly, this was a cross‐sectional study, and we were unable to analyze the changes in laboratory indicators in patients in the treatment and prognosis stages; consequently, we lack corresponding information on the prognosis of the disease. Therefore, the changes in various indicators must be analyzed after treatment of patients to provide comprehensive reference values for disease prognosis.

In conclusion, SAA and IL‐6 could be used as addition parameters to helping the distinguish of patients with COVID‐19 from healthy people, and can provide potential basis for separating patients with nonsevere and severe clinical signs.

## AUTHOR CONTRIBUTIONS

QL and FMY conceived and designed the experiments. QL, YPD, and XW performed the experiments. MMF and WL analyzed the data. QL, YPD, and FMY wrote the article.
